# Cationic double *K*-hole pre-edge states of CS_2_ and SF_6_

**DOI:** 10.1038/s41598-017-13607-6

**Published:** 2017-10-17

**Authors:** R. Feifel, J. H. D. Eland, S. Carniato, P. Selles, R. Püttner, D. Koulentianos, T. Marchenko, L. Journel, R. Guillemin, G. Goldsztejn, O. Travnikova, I. Ismail, B. Cunha de Miranda, A. F. Lago, D. Céolin, P. Lablanquie, F. Penent, M. N. Piancastelli, M. Simon

**Affiliations:** 1 Department of Physics, University of Gothenburg, Origovägen 6B, SE-412 96 Gothenburg, Sweden; 2 Department of Chemistry, Physical and Theoretical Chemistry Laboratory, Oxford University, South Parks Road, Oxford, OX1 3QZ United Kingdom; 3 Sorbonne Universités, UPMC Univ Paris 06, CNRS, UMR 7614, Laboratoire de Chimie Physique-Matière et Rayonnement, 75005 Paris Cedex 05, France; 4 Fachbereich Physik, Freie Universität Berlin, Arnimallee 14, D-14195 Berlin, Germany; 5 Synchrotron SOLEIL, L’Orme des Merisiers, Saint-Aubin, BP 48, F-91192 Gif-sur-Yvette Cedex, France; 6 Max-Born-Institut, Max-Born-Strasse 2A, 12489 Berlin, Germany; 7 Centro de Ciências Naturais e Humanas, Universidade Federal do ABC (UFABC), Av. dos Estados, 5001, 09210-580 Santo André, SP Brazil; 8 Department of Physics and Astronomy, Uppsala University, Box 516, SE-751 20 Uppsala, Sweden

## Abstract

Recent advances in X-ray instrumentation have made it possible to measure the spectra of an essentially unexplored class of electronic states associated with double inner-shell vacancies. Using the technique of single electron spectroscopy, spectra of states in CS_2_ and SF_6_ with a double hole in the K-shell and one electron exited to a normally unoccupied orbital have been obtained. The spectra are interpreted with the aid of a high-level theoretical model giving excellent agreement with the experiment. The results shed new light on the important distinction between direct and conjugate shake-up in a molecular context. In particular, systematic similarities and differences between pre-edge states near single core holes investigated in X-ray absorption spectra and the corresponding states near double core holes studied here are brought out.

## Introduction

Hollow atoms and molecules with double vacancies in inner shells are currently a subject of much interest because of their putatively enhanced chemical sensitivity especially in the molecular cases (e.g. ref.^1^). Formation of such double core holes (DCHs) in direct ionization by single photons, particularly the n = 1 (*K*) shells of the first-row atoms like C, N and O and the n = 2 (*L*) shells of the second-row atoms like S and Cl has been examined only recently^2–8^, whereas DCHs in the *L*-shells of second-row atoms have been known for some time from measurements of *KLL* Auger spectra (e.g. ref.^9^). DCH formation upon the absorption of several high-energy photons has also been reported only recently^10–16^, using intense light of an X-ray Free Electron Laser (FEL), the Linac Coherent Light source (LCLS) at Stanford.

Detection of DCHs formed by single-photon ionization became possible thanks to the availability of high-brilliance third-generation synchrotron radiation sources and to the development of a highly efficient multi-electron coincidence technique based on a magnetic bottle^2–8,17^, which is vital for the detection of electrons which can share the excess energy arbitrarily. In contrast, at FELs, the formation of double core holes utilizes high-power radiation pulses of a few femtoseconds duration which can lead to the absorption of more than one photon by the sample and hence sequential emission of the two core electrons at well defined energies before secondary decay processes set in^10–16^, principally allowing for a single channel detection scheme. Although these techniques have proved to be very informative for the characterization of double-core-hole states, they are also demanding due to their complexity and, additionally in the case of the FEL, due to the very limited amount of measuring time available at these rare facilities. A simpler experimental method would be desirable, capable of overcoming the difficulty posed by the low cross sections of the double-core-hole channels compared to the main single-hole channels (several orders of magnitude)^5,8^.

Very recently, a new, high-brilliance beam line operating in the hard X-ray region and providing high photon flux with good energy resolution has become operational at the synchrotron radiation facility SOLEIL, Paris, France^18,19^. This infrastructure has given new impulse to studies of the dynamics of photoexcitation and photoionization of low density matter, in particular of atoms and molecules in the gas phase. For instance, new phenomena such as the Auger Doppler effect^20^ and electronic state-lifetime interference^21–23^ have been observed.

In this work we employ this new infrastructure to investigate essentially unexplored DCH pre-edge states, i.e. final states based on double vacancies in the *K*-shells with one of the two electrons excited to an unoccupied valence orbital, in two molecules containing the second-row element S, i.e. CS_2_ and SF_6_. C and S *K*-edges of CS_2_ and S and F *K*-edges of SF_6_, have been investigated. These rather exotic pre-edge states with two core holes and one excited electron, sometimes referred to as core shake-up states^24^ or super shake-up states^25^, were originally observed in triple-electron coincidence events^2,26^ accumulated at a fixed photon energy, and are located at ionization energies below the DCH continuum states. They must exist quite generally, involving either the same^2,26^ or different core orbitals^24,27^. The energies of these pre-edge states, like the energies of bare DCH states, are expected to be more sensitive to differences in their chemical environment than are single core holes on the same atoms^1^. Instead of the multi-electron coincidence technique, which reveals the target states with limited resolution, we use in the present work a conventional single-electron energy analyzer, which offers higher energy resolution. This technique is suitable for the study of DCH pre-edge states where just one of the two core electrons is released into the continuum at a well-defined kinetic energy, in contrast to double core ionization where the two emitted photoelectrons can share the excess energy arbitrarily and therefore require coincidence detection (see e.g. refs^2,5^). The energy of the electron released in the formation of a DCH pre-edge state is equal to the difference between the energy of the state under investigation and the photon energy, which can be chosen so that other electrons, released subsequently, do not interfere. This technique has recently been demonstrated in the case of K^−1^ L^−1^ V states of argon^24^ and K^−2^ V states in neon^28^. We here apply it to molecules and present calculations of the relative intensities of qualitatively different shake up processes involved.

**Figure 1:**
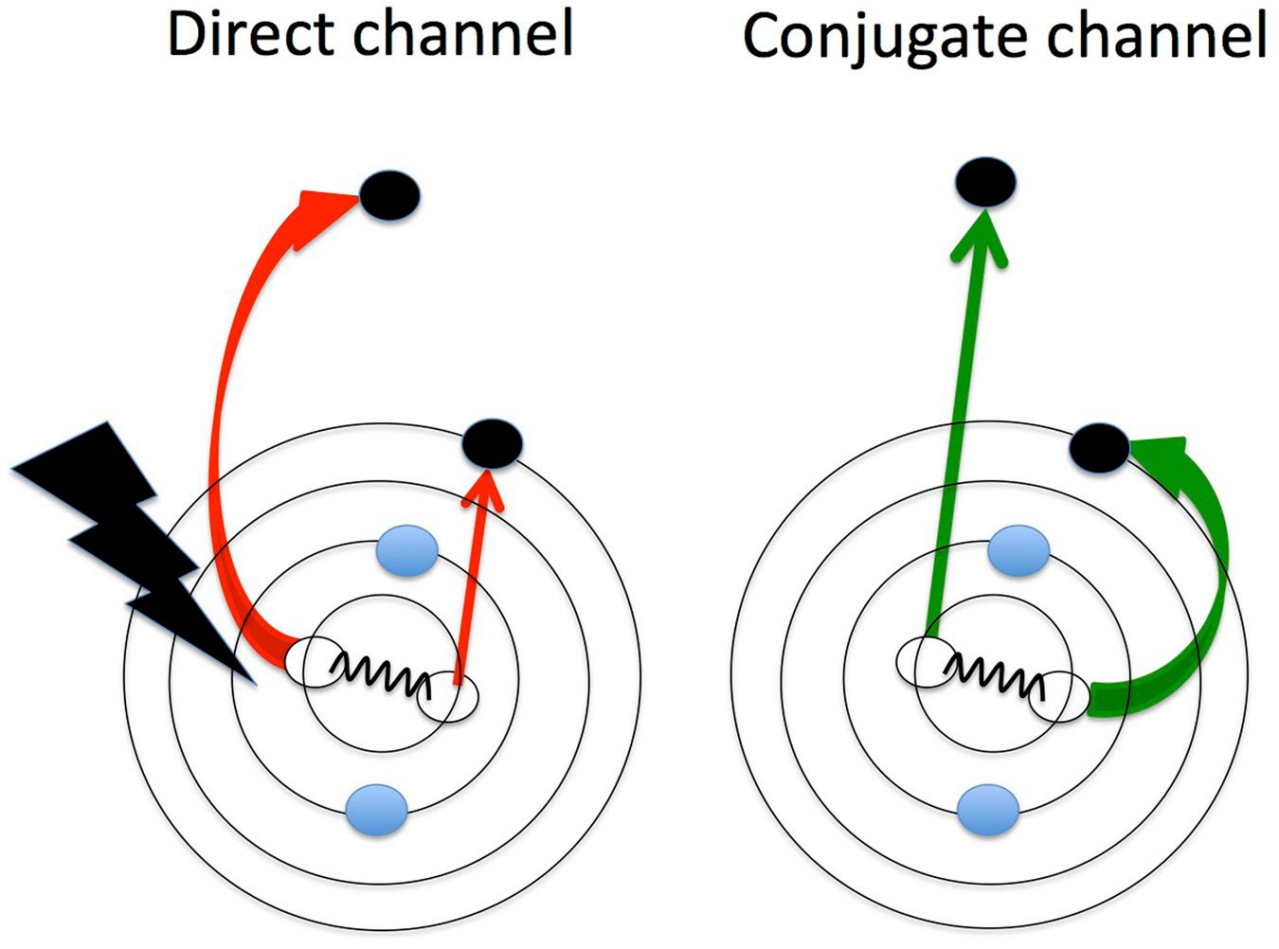
Schematic illustration of the two channels contributing to single photon *K* ^−2^ V processes. The red (green) arrows represent the direct (conjugate) path. The curved arrows correspond to photo-excitation processes. The straight arrows correspond to shake processes.

In a simplified picture with just two active electrons these pre-edge states can be thought of as being formed in two different ways upon absorption of a single photon. Either one of the two core electrons is ejected into the continuum while the second electron is shaken up into an unoccupied valence orbital by a monopole transition, or one of the two core electrons is excited into an unoccupied valence orbital in a dipole transition while the second core electron is shaken off. The first case is referred to as a *direct* shake-up process whereas the second case is denoted as a *conjugate* shake-up process (see e.g. refs^29–31^). It is important that in conjugate shake-up, where an unoccupied orbital is populated upon photon absorption, dipole selection rules govern the symmetries of the final states, just as in the formation of pre-edge resonances based on single core holes, whereas in direct shake-up the excitation step is a monopole process and the state formed must have the same symmetry as the initial core hole. Figure 1 displays a schematic of the two processes where dipolar excitations are represented by curved arrows and shake processes by straight arrows; while red arrows pertain to the direct channel, green arrows refer to the conjugate channel. The strengths of the different paths are governed, according to theory^26,32^, by two antagonistic factors whose effect may vary from one system to another but remain of comparable magnitude. This is one of the main characteristics of these DCH pre-edge processes^26,32^. The present work aims at an in-depth analysis of the relative position and intensity of the pre-edge resonances in the presence of double core holes compared with the more familiar situation where a single core hole is formed. For this purpose, our data are systematically compared with conventional X-ray absorption spectra. Similarities and differences are discussed, which provide information on the nature of the resonances and of the structure of the molecular potential.

## Results and Discussion

In what follows, *K* ^−2^ V states are investigated experimentally for the C and S *K*-edges of CS_2_ and for the S and F *K*-edges of SF_6_, and are directly compared to theoretical modelling. The binding energies of these states are calculated using a Hartree-Fock and a post Hartree Fock (Møller Plesset at second order level of perturbation, MP2) code from the GAMESS package. In addition, Density Functional Theory (DFT) calculations are performed for core-hole formation on C and F atoms. In Table 1 the experimental binding energies are reported and compared with the theoretical results obtained with the different methods. In the following, we will discuss the two molecules separately and the two edges for each of them.

**Table 1:** Experimental and theoretical binding energies (eV) of double core hole states in CS_2_ and SF_6_. The assignments are based on leading configurations.

Methods					
	Exp	HF	MP2	CI	DFT
C*S_2_					
K^−2^ $(3{\pi }_{u}^{\ast })$	635	637.99	636.04	637.23	634.60
K^−2^ $(7{\sigma }_{g}^{\ast })$	639	644.70	637.82	642.32	639.13
K^−2^	—	654.52	650.32	653.05	650.70
CS*_2_					
K^−2^ $(5{\pi }^{\ast })$	5148	5148.38	5148.14	5148.21	—
K^−2^ $(12{\sigma }^{\ast })$	(?)	5150.48	5149.48	5150.05	—
K^−2^	—	5162.10	5161.84	5161.66	—
S*F_**6**_					
K^−2^ $(6{{\rm{a}}}_{1g}^{\ast })$	5168.5	5173.05	5168.56	5170.14	—
K^−2^ $(6{{\rm{t}}}_{1u}^{\ast })$	5171	5175.52	5171.62	5173.18	—
K^−2^	—	5189.44	5186.74	5186.42	
SF*_6_					
K^−2^ $(14{{\rm{a}}}_{1}^{\ast })$	1478.5	1476.30	1476.04	1476.84	1473.61
K^−2^ $(15{{\rm{a}}}_{1}^{\ast })$	1485	1482.95	1483.14	1482.72	1480.49
K^−2^ (10e^*^)	1488	1483.80	1484.91	1484.30	1482.60
K^−2^	—	1492.28	1492.27	1491.77	1490.00

### Carbon *K*^−2^ V states of CS_2_

**Figure 2:**
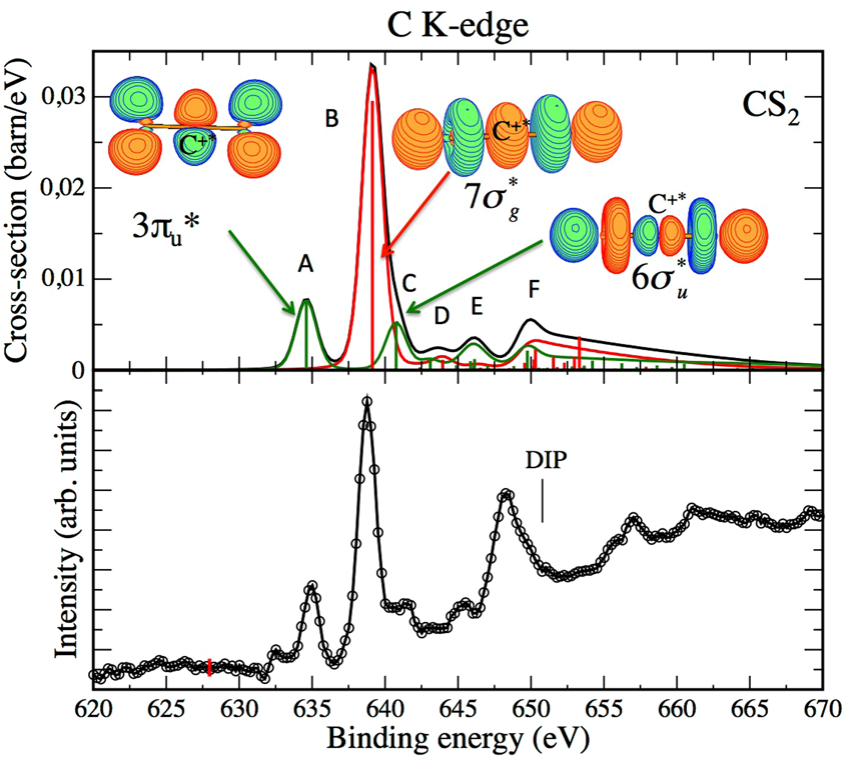
Experimental (lower panel) and theoretical (upper panel) *K* ^−2^ V spectrum of C^*^S_2_. The theoretical spectrum is based on DFT/B3LYP binding energies (see Table 1). The main peaks are illustrated by electronic iso-density plots. Red lines: direct contributions; green lines: conjugate contributions; black curve: incoherent sum of direct and conjugate contributions.

An electron spectrum of CS_2_ was obtained at the lowest possible photon energy of the GALAXIES beam line, i.e. 2.3 keV, and is shown in the lower panel of Fig. 2 on the binding energy scale. The experimental data consist of two sharp peaks, the most intense being located around 639 eV and a less intense one around 635 eV. Weaker features at around 642 eV and 645 eV and a more intense structure at around 648 eV, just below the theoretical C1s^−2^ threshold (DIP) at 650.70 eV (calculated at the DFT/B3LYP level) are also present. From this experimental CS_2_ spectrum, we subtracted a linear background so that the background is constant below the first peak.

The theoretical spectrum displayed in the upper panel of Fig. 2 reproduces the experimental spectrum very well, in terms of both relative intensities and energies. It has been obtained by a home-made post-Hartree Fock Configuration Interaction program. Its energy scale has been calibrated relative to the DFT/B3LYP value of the *K* ^−2^ $(3{\pi }_{u}^{\ast })$ resonance of CS_2_ (634.60 eV). We stress that our theoretical methods are capable of calculating very small differential cross sections, which is essential in this context. At the same time our experimental apparatus can detect the corresponding very weak features. Table 1 summarizes the theoretical binding energies for all edges of CS_2_ investigated here for direct comparison to our experimental results, where the states are labeled based on their leading configuration.

According to Wight and Brion^33^, in the case of CS_2_ the ground state electron configuration can be denoted as: ${{\rm{S}}}_{1s}^{4}\,{{\rm{C}}}_{1s}^{2}\,{{\rm{S}}}_{2s}^{4}{{\rm{S}}}_{2p}^{12}{(5{\sigma }_{g})}^{2}{(4{\sigma }_{u})}^{2}{(6{\sigma }_{g})}^{2}5{\sigma }_{u}^{2}2{\pi }_{u}^{4}2{\pi }_{g}^{4}\,\,$ and the lowest unoccupied molecular orbital (LUMO) is $3{\pi }_{u}^{\ast }$. On that basis peak A, at the lowest binding energy in the spectrum, is attributed to the K^−2^ $(3{\pi }_{u}^{\ast })$ resonance. It is entirely due to the conjugate path, as is expected from symmetry considerations for every state of *ungerade* symmetry. The corresponding iso-density plot illustrates the anti-bonding ${\pi }^{\ast }$ symmetry of the orbital, localized perpendicularly to the S=C=S internuclear axis. An analysis based on Löwdin’s populations, which are summarized in Table S1 of the Supplementary Materials, shows for the K^−2^ $(3{\pi }_{u}^{\ast })$ resonance that the density is partitioned between the p*xy* orbitals of the carbon (19%) and the sulphur (80%) atoms. The term value (TV) for peak A is 16.09 eV, much larger than the TV for the K^−1^ $(3{\pi }_{u}^{\ast })$ resonance that is observed only 7.00 eV below the single core-hole threshold in the C-*K* edge NEXAFS spectra of CS_2_^33^. We note that the mean square radius value is almost the same for the *K* ^−2^ $(3{\pi }_{u}^{\ast })$ and *K* ^−1^ $(3{\pi }_{u}^{\ast })$ resonances (see Table S1 of the Supplementary Material). This results from two antagonistic effects, orbital shrinking due to the presence of the second core hole and electronic expansion towards the neighboring sulphur atoms (as reflected in Löwdin’s populations) due to the antibonding character of the valence orbital. The significant difference between the *K* ^−1^ and *K* ^−2^ TVs can be explained by a substantial increase of the effective charge felt by the electron in the *K* ^−2^ $(3{\pi }_{u}^{\ast })$ case because of the larger penetrability of the valence orbital in the presence of a double core hole.

Peak B at around 639 eV corresponds essentially to the *K* ^−2^ $(7{\sigma }_{g}^{\ast })$ resonance populated by a pure direct shake-up process as is expected for every state of *gerade* symmetry, which demonstrates a great asset of *K* ^−2^ V spectroscopy: to reveal dipole-forbidden transitions not accessible in conventional X-ray absorption spectroscopy, especially when vibronic coupling is absent^34^. Indeed, in the C *K*-edge NEXAFS spectra, the 1s→$(7{\sigma }_{g}^{\ast })$ line does not appear.

The shoulder C on the high-binding-energy side of peak B can be assigned, according to our calculations, to the 1s→$6{\sigma }_{u}^{\ast }$ resonance populated by a pure conjugate shake-up process. The density plots included in Fig. 2 and Löwdin’s populations (see Table S1) give s and d populations for the $7{\sigma }_{g}^{\ast }$ resonance and p population for the $6{\sigma }_{u}^{\ast }$ resonance around the C atom, while around the S atoms p and d populations are observed for both resonances. The *K* ^−1^ $(6{\sigma }_{u}^{\ast })$ resonance, even though dipole-allowed, has not been observed in NEXAFS spectra, neither below nor above the *K* ^−1^ threshold. It is interesting to note that in the centro-symmetric N_2_ case, Carniato *et al*.^35^ found the *K* ^−2^ ${\sigma }_{u}^{\ast }$ resonance below the *K* ^−2^ threshold while the ${K}^{-1}{\sigma }_{u}^{\ast }$ resonance was observed and calculated above the *K* ^−1^ ionization threshold in the associated single core hole spectra. Such displacement effects are expected to be less-pronounced in CS_2_ since the CS_2_ $7{\sigma }_{g}^{\ast }$ and $6{\sigma }_{u}^{\ast }$ valence orbitals are not such strongly anti-bonding orbitals as is the ${\sigma }^{\ast }$ orbital of N_2_, while the double core hole potential is less attractive in CS_2_ than in N_2_. According to our DFT calculations the ${K}^{-2}(7{\sigma }_{g}^{\ast })$ and ${K}^{-2}(6{\sigma }_{u}^{\ast })$ resonances are pushed 7 eV further away from the *K* ^−2^ threshold than are the ${K}^{-1}(7{\sigma }_{g}^{\ast })$ and ${K}^{-1}(6{\sigma }_{u}^{\ast })$ resonances from the *K* ^−1^ threshold. The high-binding-energy region (from 642 eV onward) reflects additional features which are most likely related to Rydberg states and/or multi-electron excitations. A tentative analysis of the main components is reported in Table S1. However, due to the complexity of this region, a one-to-one assignment would not be reliable, and is not attempted.

### Sulphur *K*^−2^ V states of CS_2_

**Figure 3:**
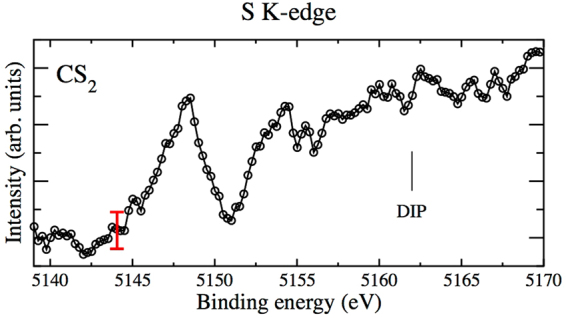
Experimental sulphur *K* ^−2^ V spectrum of CS_2_.

For the CS_2_ molecule, an experimental spectrum has also been recorded in the binding energy region corresponding to *K* ^−2^ V states with double core vacancies on the sulphur atom using a photon energy of 5.9 keV. It is shown in Fig. 3, where a first clear peak is observed at a binding energy of about 5148 eV, and where a second peak possibly emerges from the background (of unknown origin) at around 5153 eV. Again, we subtracted a linear background so that the background is constant below the first peak.

In order to assign this spectrum, we performed calculations in this binding energy region, which are presented in Table 1 and where two *K* ^−2^ V resonances have been identified. The first one corresponds well to the excitation of a sulphur 1s electron to the LUMO 5*π* (labeled in C_∞*v*_ symmetry and corresponding to $3{\pi }_{u}^{\ast }$ in D_∞*h*_) with a binding energy of around 5148 eV, in very good agreement with the experiment. Electronic iso-density plots (not shown) confirm the *π* nature of the valence molecular orbital, where the electronic density is unequally distributed between the two sulphur atoms. The second resonance could correspond to the excitation to the $12{\sigma }^{\ast }$ valence orbital (corresponding mainly to the $7{\sigma }_{g}^{\ast }$ molecular orbital in D_∞*h*_ plus a small mixing with the $6{\sigma }_{u}^{\ast }$ orbital), though its calculated binding energy of around 5150 eV is somewhat lower compared to the experimental value considered. Associated resonances have been observed in sulphur 1s NEXAFS spectra^36^ where the first two major peaks, which are 3 eV apart, have been similarly assigned to S 1s →$3{\pi }_{u}^{\ast }$ and S 1s(→${\sigma }_{g}$ + ${\sigma }_{u}$) excitations. However, in view of the experimental width of the first peak structure of more than 3 eV (FWHM) and its asymmetric shape versus the theoretical energy spacing of the two resonances of about 2 eV, it seems also possible that this spectral feature comprises two states, where in its lower part around 5146 eV the *K* ^−2^5π resonance and in its higher energy part around 5148 eV the *K* ^−2^12σ resonance might be located. This assignment would perfectly fit to the calculated splitting of about 2 eV as well as to the intensities at the carbon edge where the ${K}^{-2}3{\pi }_{u}$ (conjugate shake) resonance is less intense that the ${K}^{-2}7{\sigma }_{g}$ (direct shake) resonance. In addition, the absolute energy values would still be in very good agreement with the theoretical results bearing in mind the present experimental accuracy in absolute energy calibration.

### Sulphur *K*^−2^ V states of SF_6_

**Figure 4:**
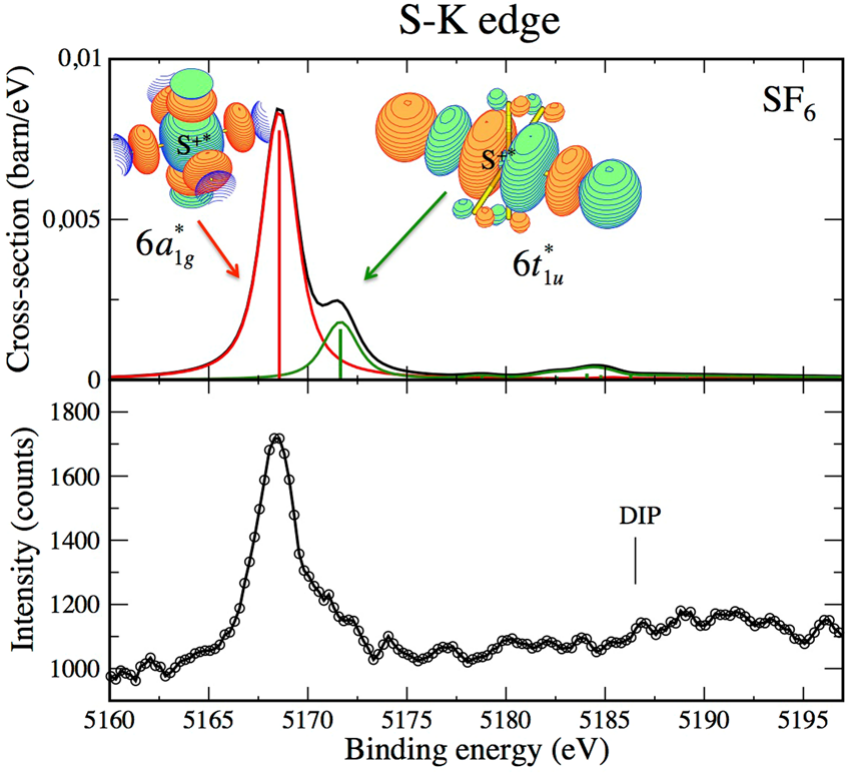
Experimental (lower panel) and theoretical (upper panel) sulphur *K* ^−2^ V spectra of SF_6_. Red lines reflect the direct contributions and green lines reflect conjugate contributions. The black curve represents an incoherent sum of the direct and conjugate contributions.

Figure 4 shows the 5160–5195 eV binding energy range of an electron spectrum of S^*^F_6_ which was measured at a photon energy of 5.9 keV. This spectrum contains an intense and asymmetric peak located around 5168.5 eV with a pronounced shoulder at around 5171 eV. In order to assign this spectrum we make use again of calculations and build on the notation of the ground state electron configuration of SF_6_ known from the work of Dehmer^37^: ${{\rm{S}}}_{1s}^{2}$ ${{\rm{F}}}_{1s}^{12}$ ${{\rm{S}}}_{2s}^{2}$ ${{\rm{S}}}_{2p}^{6}$ ${{\rm{F}}}_{2s}^{12}$ $5{{\rm{a}}}_{1g}^{2}$ $1{{\rm{t}}}_{2g}^{6}$ $4{{\rm{t}}}_{1u}^{6}$ $1{{\rm{t}}}_{2u}^{6}$ $3{{\rm{e}}}_{g}^{4}$ $1{{\rm{t}}}_{1g}^{6}$ $5{{\rm{t}}}_{1u}^{6}$. Table 1 summarizes the theoretical binding energies for all edges of SF_6_ investigated here for direct comparison to our experimental results, where the states are labeled based on their leading configuration.

Our calculations suggest that the first peak corresponds to a direct shake-up contribution and is associated with the *K* ^−2^6a_1*g*_ resonance, where the $6{{\rm{a}}}_{1g}^{\ast }$ orbital is the LUMO. The second peak corresponds to a conjugate shake-up contribution and is associated with the ${K}^{-2}6{t}_{1u}^{\ast }$ resonance, where $6{{\rm{t}}}_{1u}^{\ast }$ is the first unoccupied anti-bonding molecular orbital of t_1*u*_ symmetry. This orbital consists mainly of sulphur 3p and fluorine 2p atomic orbitals, as reflected in the iso-electronic density plots included in the upper panel of Fig. 4. Again, as expected from selection rules, direct contributions select *gerade* states and conjugate contributions select *ungerade* states, so that the two contributions have to be added incoherently. There is no indication in the experimental spectrum of any Rydberg series.

The overall agreement between experiment and theory is good in terms of both energy positions and relative intensities. Also, the main features of the *K* ^−2^ V S^*^F_6_ spectrum can be compared to those of sulphur 1s NEXAFS spectra, in which the $1{a}_{1g}\to 6{t}_{1u}^{\ast }$ transition is identified as the main resonance while Rydberg series seem also be absent^37–39^.

In addition, our theoretical spectrum suggests a fairly broad and weak structure just below the DIP which can be assigned to resonances of t_2*g*_ and e_*g*_ symmetry. These resonances might be associated with the formation of *K* ^−2^2t_2*g*_ and *K* ^−2^4e_*g*_ states. Equivalent *K* ^−1^2t_2*g*_ and *K* ^−1^4e_*g*_ states were observed in sulphur *L* ^−1^ V spectra where they appeared as sharp structures about 20 eV from the main peak^37–39^. In the present sulphur *K* ^−2^ V spectra the weak 1a_1*g*_→ 2t_2*g*_/4e_*g*_ structures are suggested to be shifted towards the main 1a_1*g*_→  6a_1*g*_ line, which appears as a commonly encountered characteristic in *K* ^−2^ V spectra^25,35^.

Although, in the light of the present experimental resolution, any putative vibrational progression must be expected to be masked by the very short *K* ^−2^ lifetime, a large deformation of the initial geometry is likely to occur in the presence of two inner-shell holes. A detailed vibrational analysis, summarized in Table S2 of the Supplementary Materials, following simultaneous S 1s ionization and promotion of a S 1s electron to a 6a_1*g*_ orbital suggests a large gradient associated with the symmetric stretching (A_1*g*_) mode (*v* = 731 cm^−1^). The six relaxed S-F interatomic distances in the final state increase significantly (1.743 Å) compared to the neutral ground state (1.579 Å). The ${{\rm{\Delta }}}_{tot}^{FC}$ (FWHM) of the Franck-Condon profile is estimated as ≈0.9 eV (see Table S2) assuming that the frequency in the final state and the neutral state are the same. In fact the symmetric mode of concerted S-F bond elongation is subject to frequency softening by nearly 40% (*v* = 445 cm^−1^), which calls into question our approximate model of the same pool of frequencies for the initial neutral ground state and the *K* ^−2^ V final state. Taking this aspect into account, ${{\rm{\Delta }}}_{tot}^{FC}$ (FWHM) of the Franck-Condon profile is estimated to be slightly larger, namely 1.1 eV, which is still close to 1 eV and therefore validates our approximation in the present context.

In the case of the 6t_1*u*_ mode, additional excitation of degenerate antisymmetric E_*g*_ vibrational modes takes place with similar width of the Franck-Condon profile to the one calculated for the symmetric stretching mode. This corresponds to a large deformation of the SF_6_ geometry with the shortening of the S-F bonds by 11 pm in the equatorial plane and elongation by 50 pm of the axial S-F bonds. In any case, these rich vibrational progressions make the structure much broader than can be explained by the experimental resolution (for sulphur, the double core hole lifetime is assumed 3 × Γ_*S*1*s*_ = 1.5 eV and the experimental resolution is set to 1.1 eV).

### Fluorine *K*^−2^ V states of SF_6_

**Figure 5:**
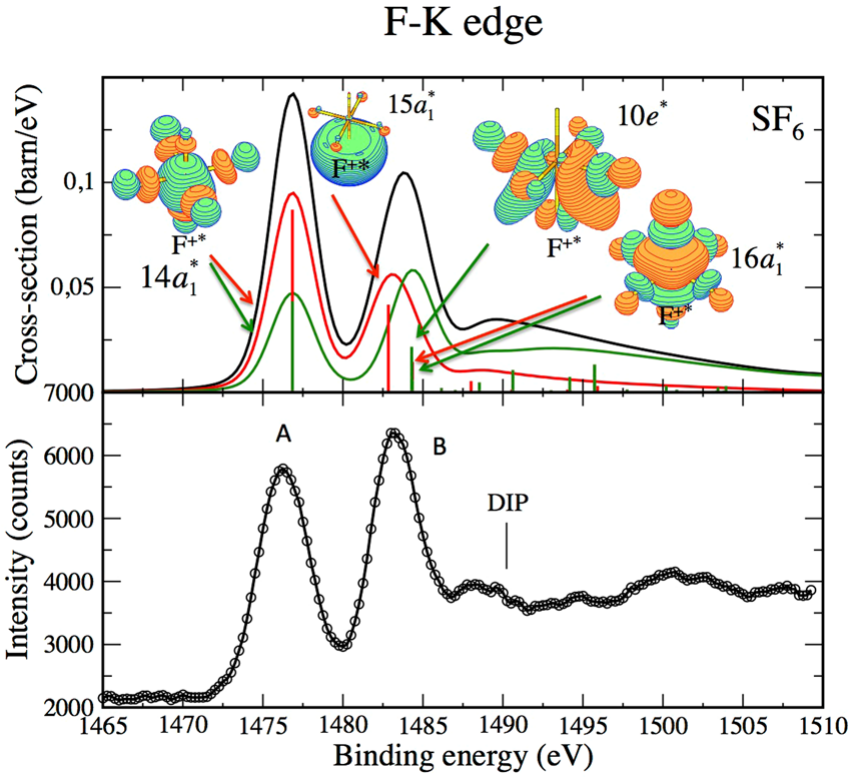
Experimental (lower panel) and theoretical (upper panel) *K* ^−2^ V spectra of ${{\rm{SF}}}_{6}^{\ast }$. The position of the theoretical low-lying band is set to 1476.04 eV (see Table 1, HF-MP2).

An experimental spectrum has been obtained in the region of the fluorine *K*-edge at the lowest possible photon energy of the GALAXIES beam line (2.3 keV). It is shown in Fig. 5 and reflects two strong peaks; A around 1478.5 eV and B around 1485 eV, and a weaker and broader band around 1490 eV binding energy. In the fluorine case our core ionization vibrational analysis (see Table S2 of the Supplementary materials which reports only gradients greater than 10 eV/Å) shows a large gradient associated with the triply degenerate F_1*u*_ symmetric stretching mode at *v* = 890 cm^−1^ in addition to a strong activation of the symmetric (A_1*g*_) and the doubly-degenerate (E_*g*_) modes as observed for S-*K* ^−2^ V. This leads to a large FWHM of the two main peaks at 1476 eV and 1484 eV, reproducing fairly well the experimental spectral broadening (for fluorine, the double core hole lifetime is assumed 3 × Γ_*F*1*s*_ = 0.75 eV and the experimental resolution is set to 1.1 eV).

The theoretical spectrum displayed in the top panel assigns peak A to a unique *K* ^−2^ V state where V is the first unoccupied orbital of a_1_ symmetry according to the C_4*v*_ group $(14{{\rm{a}}}_{1}^{\ast })$. Going from neutral SF_6_ to ${{\rm{SF}}}_{6}^{+\ast }$ with a double core vacancy lowers the symmetry from O_*h*_ to C _4*v*_. This state can be reached from both the direct and the conjugate pathways through the F 1s→$6{{\rm{a}}}_{1g}^{\ast }$ transition. Indeed the fluorine s orbitals split into orbitals of t_1*u*_, e_*g*_ and a_1*g*_ symmetry in an octahedral configuration, so that direct contributions associated to monopolar selection rules are allowed through the a_1*g*_→ a_1*g*_ component while conjugate contributions associated to dipolar selection rules are allowed trough the t_1*u*_→ a_1*g*_ component. Direct and conjugate contributions leading to the ${K}^{-2}14{a}_{1}^{\ast }$ state might interfere. We did not take into account such interference effects, but present the cross section as an incoherent superposition (black line) of direct and conjugate contributions.

In region B the theoretical calculations reveal a pure direct contribution leading to the ${K}^{-2}15{a}_{1}^{\ast }$ state, a pure conjugate contribution leading to the *K* ^−2^10e^*^ state where 10e^*^ is the first unoccupied molecular orbital of e symmetry, and a mixed direct/conjugate contribution leading to the ${K}^{-2}16{{\rm{a}}}_{1}^{\ast }$ state. Again interference effects were disregarded in the latter case.

The weaker structure observed experimentally and theoretically around 1490 eV is likely to correspond to one of the shape resonances observed in the *K* ^−1^ V X-ray absorption spectrum^40^ that might be pushed below the ionization threshold in the *K* ^−2^ V spectrum.

## Conclusions

By capitalising on new experimental possibilities using a comparatively simple electron detection technique in combination with X-rays in the 2–13 keV (tender) range, we have been able to measure, the *K* ^−2^ *V* spectra from double vacancies on the carbon and sulphur atoms in CS_2_ and the sulphur and fluorine atoms in SF_6_ with unprecedented ease and high resolution. The spectra have been interpreted in detail based on Post-Hartree-Fock (CI-SDT) calculations. A comparison of conjugate *K* ^−2^ V and *K* ^−1^ V spectra for an atom-specific *K*-edge shows close similarities when the holes are on the central atoms. A crucial difference is that while conventional X-ray absorption spectra near single-core-hole edges often have clear structures which persist well above the edges, this does not seem to be the case in the conjugate shake up part of the *K* ^−2^ V spectra. One possible explanation could be that above-threshold features due to shape resonances are pushed below threshold and weakened by the different nature of the molecular potential from double core vacancies. Another possibility might be that above-threshold features cannot be detected efficiently by the present technique, since they cause the emission of two electrons so that their exact energy position is more difficult to determine. Moreover, the expected energy sharing between the two outgoing electrons causing a slow and a fast one is probably similar to that of the direct shake-off process of double ionization; this might hamper a efficient discrimination of the two processes.

Finally, we note that the absolute energies of the double core hole states calculated here are accurate to within 1 eV. Because the energy gaps between the bare double core holes and the principal *K* ^−2^ V states are calculated even more accurately, one can in principle use the combination of the present kind of experiment and numerical calculations to determine the positions of double-core-hole continuum states.

## Methods

### Experimental

The experiments were carried out at the GALAXIES beam line^18^ of the synchrotron radiation facility SOLEIL in Paris, which covers the 2–13 keV energy range, and offers the capability to form DCH states involving deep inner-shells. The present investigations utilised the permanent HAXPES (hard x-ray photoelectron spectroscopy) end station of this beam line which comprises a commercially available, hemispherical electrostatic electron kinetic energy analyser (Scienta EW4000), optimised for high-resolution studies of high kinetic energy electrons^19^. This spectrometer is placed in the plane of the electric field vector of the linearly polarised radiation, detecting primarily electrons at 0° with respect to the polarisation axis. For the present measurements, it was operated using a 0.8 mm curved slit and a pass energy of 500 eV which results in an analyser resolution of about 1 eV. Combining this value with the photon energy bandwidth (about 200 meV at 2.3 keV and about 800 meV at 5.9 keV) results in an overall resolution of about 1 eV for the F *K* ^−2^ V and C *K* ^−2^ V measurements, and about 1.3 eV for the S *K* ^−2^ V measurements. The kinetic energy scale of the spectrometer was calibrated by measuring the *KMM* and *LMM* Auger spectrum of Ar akin to ref.^24^. With the calibrated electron spectrometer at hand, we could also measure the photon energies used by recording the well-known Ar 1*s* ^41^ and Ar 2*p* photoelectron spectra^42^. Overall, the binding energies for the DCH pre-edge states reported are determined with a systematic uncertainty of about 0.7 eV. Depending on the signal strength, the data acquisition time was typically on the order of 2–12 hours per spectrum.

Since the Scienta EW4000 analyser utilizes a 2D MCP/CCD detector system which typically gives more than one count for one detected electron, the statistical scattering of the data is not directly given by the square root of the count ${N}_{i}$, which represents the count at the energy ${x}_{i}$ given by the detector system. To overcome this shortcoming, the real count $\alpha {N}_{i}$ can be estimated by determining the scaling factor *α* for the entire spectrum. For this purpose, the unstructured background is fitted by a fit function $f(x)=a{x}^{2}+bx+c$, assuming that

1 $${\chi }^{2}=\sum _{i}\frac{\alpha {N}_{i}-f({x}_{i})}{\sqrt{\alpha {N}_{i}}}=n-p-\mathrm{1,}$$

with *n* being the number of data points and *p* = 3 the number of free parameters in the fit model. In this way, the resulting $\alpha {N}_{i}$ is the most likely count relating to the observed statistical scattering of the background. We note that similar *α*’s were obtained for the different spectra with $1/\alpha =16\pm 2.5$.

In the case of SF_6_, both spectra reflect an intense signal on a weak background, and the vertical axes in Figs 4 and 5 represent the estimated real count $\alpha {N}_{i}$. Accordingly, the statistical scattering can be obtained as ${\rm{\Delta }}\alpha {N}_{i}=\sqrt{\alpha {N}_{i}}$.

In contrast, in the case of CS_2_ (cf. Figs 2 and 3) the raw signals are on a comparatively strong and non-constant background which define the statistical scattering of the data points. The data displayed in Figs 2 and 3 are background corrected. In these cases the statistical scattering ${\rm{\Delta }}\alpha {N}_{i}$ defined by the background can be considered being constant and is indicated by red vertical bars.

We also note that for reasons of presentation, the spectra shown in Figs 2 to 5 are slightly smoothed by averaging over 3 neighboring data points. This corresponds to an averaging over 500 meV which is well below the experimental resolution.

SF_6_ gas was commercially obtained with a stated purity of better than 99%. CS_2_, which at room temperature is a volatile liquid of sufficiently high vapour pressure, was purified from air contamination by several freeze-thaw cycles, and the purity of this sample was verified by recording on-line S 1*s* ^43^ and C 1*s* photoelectron spectra known in the literature (see e.g. ref^44^. and refs therein).

### Theory

The *K* ^−2^ V intensities have been calculated at a Post Hartree-Fock Configuration Interaction (CI-SDT) level of theory as already described in Refs^25,26,28,32^. The initial and final states are built from a unique set of Hartree-Fock orthogonal molecular orbitals (MOs) optimized for the associated singly core-ionized *K* ^−1^ molecular ions. This optimization is carried out using large aug-cc-pCV5Z basis sets augmented by $\mathrm{(3}s,\,3p,\,3d)$ diffuse orbitals added in order to better describe the Rydberg state energy region. The ground state CI wave function is expanded over single and double valence excitations and also single core excitations that have minor weights but are the only ones likely to produce non zero direct contributions. In order to balance the description of initial and final states these latter are expanded over single (2h-1p), double (3h-2p) and triple (4h-3p) excitations.

The Configuration Interaction calculations reproduce most of the correlation/relaxation effects upon core ionization and provide reasonable relative energy positions of the various bands. In order to determine the absolute energy position of the spectrum, calculations of the *K* ^−2^ V core binding energies were performed at a Hartree-Fock/MP2/DFT level of theory coupled with Douglas/Kroll scalar relativistic effects of the third order within the GAMESS(US) package. Large aug-cc-pCV5Z Gaussian basis for carbon, sulphur and fluorine were used in that procedure in order to describe relaxation and differential correlation effects, as well as core binding energies with a high level of accuracy.

Cross sections are obtained in the dipole approximation from the transition amplitudes ${T}_{fo}(\vec{k})$ calculated in the length gauge. The partial differential cross sections corresponding to a final ionic state f and a photoelectron of momentum $\overrightarrow{k}$ recorded in the solid angle ${{\rm{\Omega }}}_{k}$ are given in atomic units by the relation:

2 $$\frac{d{\sigma }_{f}(\omega )}{d\varepsilon \,d{{\rm{\Omega }}}_{k}}=4{\pi }^{2}\alpha \,\frac{{(B{E}_{f}+\varepsilon )}^{2}}{\omega }\,{\rho }_{f}(\varepsilon )\langle |{T}_{fo}(\overrightarrow{k},\overrightarrow{\beta }{)|}^{2}\rangle \,\delta (\omega -(B{E}_{f}+\varepsilon );{{\rm{\Gamma }}}_{f})$$

where

3 $$|{T}_{fo}(\vec{k},\vec{\beta }{)|}^{2}=|\langle {{\rm{\Psi }}}_{f}(N)|\sum _{j\mathrm{=1}}^{N}{z}_{j}|{{\rm{\Psi }}}_{o}(N)\rangle {|}^{2}$$

${{\rm{\Psi }}}_{o}(N)$ and ${{\rm{\Psi }}}_{f}(N)$ are the wave functions for the N-electron initial and final states, respectively, *zj* are the coordinates of the individual electrons along the polarization axis, $\varepsilon =\frac{{\overrightarrow{k}}^{2}}{2}$ is the energy of the photoelectron, ${\rho }_{f}(\varepsilon )$ is the density of final states in the continuum, and BE_*f*_ are the binding energies of the final ionic states. The $\delta (\omega ;\,{\rm{\Gamma }})$ function is the Lorentzian function taking into account the lifetimes of the final ionic states. The brackets surrounding $|{T}_{fo}(\vec{k},\vec{\beta }{)|}^{2}$ indicate averaging over molecular orientations identified by the three Euler angles $\overrightarrow{\beta }$. Within the creation and annihilation operator formalism described in^45^ each final state can be written as:

4 $$|{{\rm{\Psi }}}_{f}(N)\rangle ={\hat{a}}_{\overrightarrow{k}}^{+}|{{\rm{\Psi }}}_{f}(N-\mathrm{1)}\rangle $$

where ${{\rm{\Psi }}}_{f}(N-\mathrm{1)}$ is a bound eigenstate of the residual ion, and ${\hat{a}}_{\overrightarrow{k}}^{+}$ the operator for the creation of a continuum state $|\vec{k}\rangle$. Channel couplings in the continuum and correlation between the ejected electron and the remaining ones are neglected. Introducing in the transition amplitudes (2) the product state (3) and the operator ${\hat{a}}_{K}$ that annihilates the MO $|{\phi }_{K}\rangle$ for the initial state, a separation appears^26,32^ between Direct DT_fo_ and Conjugate CT_fo_ contributions, each of them being the product of a dipolar and a monopolar (overlap) matrix element:

5 $${T}_{fo}(\vec{k},\vec{\beta })=D{T}_{fo}(\vec{k},\vec{\beta })+C{T}_{fo}(\vec{k},\vec{\beta })$$

6 $$D{T}_{fo}(\vec{k},\vec{\beta })={S}_{fK}\,{d}_{K}(\vec{k},\vec{\beta })\,{\rm{and}}\,C{T}_{fo}(\vec{k},\vec{\beta })={D}_{fK}(\vec{\beta })\,{s}_{K}(\vec{k},\vec{\beta })$$

The notation of the direct and conjugate contributions is inspired by the work of Martin and Shirley^46^ to describe *K* ^−1^ valence shake-up (*K* ^−1^ v^−1^ V) states in atomic processes. The dipolar matrix elements and overlaps are defined as:

7 $${d}_{K}(\vec{k},\vec{\beta })=\langle \vec{k}|z|{\phi }_{K}\rangle \,{\rm{and}}\,{D}_{fK}(\vec{\beta })=\langle {{\rm{\Psi }}}_{f}(N-\mathrm{1)|}\sum _{j}^{N-1}{z}_{j}|{\hat{a}}_{K}{{\rm{\Psi }}}_{o}(N)\rangle $$

and

8 $${S}_{fK}=\langle {{\rm{\Psi }}}_{f}(N-\mathrm{1)|}{\hat{a}}_{K}{{\rm{\Psi }}}_{o}(N)\rangle \,{\rm{and}}\,{s}_{K}(\vec{k},\vec{\beta })=\langle \vec{k}|{\phi }_{K}\rangle $$

As illustrated in Fig. 1, the direct contribution corresponds to a dipolar core ionization accompanied by a core shake-up, and the conjugate contribution to a dipolar core excitation accompanied by a core shake-off.

In the case of centro-symmetric molecules and when the core-holes are located on the center atom, the cross sections are either purely direct or purely conjugate. Moreover, when the initial ${\phi }_{K}$ orbital is modeled by an atomic 1s orbital, which is the approximation we have implemented in our models, simplifying selection rules appear. This situation concerns C(*K* ^−2^) in CS_2_ and S(*K* ^−2^) in SF_6_. In these cases the differential conjugate cross sections for a M_*f*_ final state are then given by equation (1) in which the molecular averaging gives:

9 $$\langle |C{T}_{{M}_{f}}(\vec{k},\vec{\beta }{)|}^{2}\rangle \,{\rho }_{f}(\varepsilon )=\frac{1}{3}|{D}_{{M}_{f}{X}_{q}}{|}^{2}\,|\langle \varepsilon |{\phi }_{K}\rangle {|}^{2}|{Y}_{00}({{\rm{\Omega }}}_{k}{)|}^{2}$$

where $|{D}_{{M}_{f}{X}_{q}}{|}^{2}=|\langle {{\rm{\Psi }}}_{{M}_{f}}(N-\mathrm{1)|}{\sum }_{j}^{N-1}{X}_{qj}|{\hat{a}}_{K}{{\rm{\Psi }}}_{o}(N)\rangle {|}^{2}$ are the dipolar matrix elements evaluated in the molecular frame, *X*_*q*_ being selected according to the value of M_*f*_. The square overlaps $|\langle \varepsilon |{\phi }_{K}\rangle {|}^{2}$ are normalized per unit energy and derived from the Stieljes procedure described below. The photoelectron angular distribution is isotropic in the laboratory frame. For direct differential cross sections molecular averaging gives:

10 $$\langle |D{T}_{{M}_{f}\mathrm{=0}}(\vec{k},\vec{\beta }{)|}^{2}\rangle \,{\rho }_{f}(\varepsilon )=\frac{1}{3}|{S}_{fK}{|}^{2}\sum _{q\mathrm{=1}}^{3}|\langle {\varepsilon }_{q}|{X}_{q}|{\phi }_{K}\rangle {|}^{2}|{Y}_{10}({{\rm{\Omega }}}_{k}{)|}^{2}$$

Each photoelectron of energy *ε* may have three different orientations in the molecular frame, so that the three terms $|\langle {\varepsilon }_{q}|{X}_{q}|{\phi }_{K}\rangle {|}^{2}$ must be taken into account.

In the general case, Racah algebra can be implemented and an atomic-like relation can be derived^47^:

11 $$\frac{d{\sigma }_{f}(\omega )}{d\varepsilon \,d{{\rm{\Omega }}}_{k}}=\frac{1}{4\pi }\frac{d{\sigma }_{f}(\omega )}{d\varepsilon }\mathrm{[1}+{\beta }_{f}{P}_{2}({\rm{c}}{\rm{o}}{\rm{s}}{\theta }_{k})]$$

Asymmetry parameters are easily obtained in the particular cases discussed just above, *β*_*f*_ = 0 for conjugate terms, and *β*_*f*_ = 2 for direct terms, as indicated in equations (8) and (9) respectively. Taking into account the angular acceptance of the analyzer, that is ${\theta }_{0}=\pm 20$ degrees, gives an angular factor of $\frac{1-\,\cos \,{\theta }_{0}}{2}\sim 0.03$ for the conjugate term and $\frac{1-{\cos }^{3}{\theta }_{0}}{2}\sim 0.085$ for the direct term. The theoretical photoelectron spectra in these centro-symmetric cases are displayed in Figs 2 and 5 for respectively C-*K* edge in CS_2_ and S-*K* edge in SF_6_. They give absolute cross sections. We can underline that the integral cross sections derived in the C-*K* edge is about 0.7 barn for both the conjugate $3{\pi }_{u}^{\ast }$ and direct $7{\sigma }_{g}^{\ast }$ resonances, in excellent agreement with the experimental and theoretical values obtained at the same photon energy for the C-*K* edge in the CO_2_ molecule^25^.

Unfortunately derivation of the asymmetry parameters is very hard in the general case of non centro-symmetric core holes. In order to simulate the photoelectron spectrum obtained at the F-*K* edge in SF_6_, we have applied the same modelling as in the centro-symmetric cases. The initial *K* molecular orbital is supposed to be localized on a unique fluorine atom and a supplementary simple degeneracy factor of six is introduced in the cross section expressions. Such a modelling gives the theoretical cross sections displayed in Fig. 5. The overall agreement with experimental results is good. Only the ratio of the two major peaks deviates. This ratio is closely dependent on the asymmetry parameter. The derivation of a *β*_*c*_ = 0 parameter for pure conjugate terms can be justified through a plane wave behaviour. If such a description is used for the photoelectron, the delocalization of the core-hole apart from the center of symmetry leads to an overall phase factor that does not impact the square modulus of the pure conjugate term. The asymmetry parameter for pure direct term is no longer ${\beta }_{D}=2$, partial $\ell =0$ waves appearing also in those terms according to the plane wave approximation. The resulting decay of the *β*_*D*_ parameter moves the peak ratio in direction towards the experimental ratio. Moreover interferences may occur between conjugate and direct terms, that are out of reach in our modelling.

### Data availability

The datasets generated during and/or analysed during the current study are available from the corresponding author on reasonable request.

## Electronic supplementary material

Supplementary Materials

## References

[1] Cederbaum LS, Tarantelli F, Sgamelloti A, Schirmer J (1986). On double vacancies in the core. J. Chem. Phys..

[2] Eland JHD (2010). Double core hole creation and subsequent Auger decay in NH_3_ and CH_4_ molecules. Phys. Rev. Lett..

[3] Linusson P, Takahashi O, Ueda K, Eland JHD, Feifel R (2011). Structure sensitivity of double inner-shell holes in sulfur-containing molecules. Phys. Rev. A.

[4] Lablanquie P (2011). Properties of Hollow Molecules Probed by Single-Photon Double Ionization. Phys. Rev. Lett..

[5] Lablanquie P (2011). Evidence of Single-Photon Two-Site Core Double Ionization of C_2_H_2_ Molecules. Phys. Rev. Lett..

[6] Nakano M (2013). Single Photon K^−2^ and K^−1^K^−1^ Double Core Ionization in C_2_H_2*n*_ (n = 1-3), CO, and N_2_ as a Potential New Tool for Chemical Analysis. Phys. Rev. Lett..

[7] Hedin L (2014). N1s and O1s double ionization of the NO and N_2_O molecules. J. Chem. Phys..

[8] Hedin L (2014). Single site double core level ionization of OCS. Chem. Phys..

[9] Keski-Rahkonen O, Krause MO (1976). Energies and chemical shifts of the sulphur 1s level and the KL_2_L_3_(^1^D_2_) Auger line in H2S, SO2 and SF6. J. Electron. Spectrosc. Relat. Phenom..

[10] Young L (2010). Femtosecond electronic response of atoms to ultra-intense X-rays. Nature.

[11] Cryan JP (2010). Auger Electron Angular Distribution of Double Core-Hole States in the Molecular Reference Frame. Phys. Rev. Lett..

[12] Fang L (2010). Double Core-Hole Production in N_2_: Beating the Auger Clock. Phys. Rev. Lett..

[13] Berrah N (2011). Double Core-Hole Spectroscopy for Chemical Analysis with an Intense X-ray Femtosecond Laser. Proc. Natl. Acad. Sci..

[14] Salén P (2012). Experimental verification of the chemical sensitivity of two-site double core-hole states formed by an x-ray free-electron laser. Phys. Rev. Lett..

[15] Frasinski LJ (2013). Dynamics of hollow atom formation in intense x-ray pulses mapped by partial covariance. Phys. Rev. Lett..

[16] Mucke M (2013). Covariance mapping of two-photon double core hole states in C_2_H_2_ and C_2_H_6_ produced by an X-ray free electron laser. New J. Phys..

[17] Eland JHD (2003). Complete Two-Electron Spectra in Double Photoionization: The Rare Gases Ar, Kr, and Xe. Phys. Rev. Lett..

[18] Rueff J-P (2015). The GALAXIES beamline at the SOLEIL synchrotron: inelastic X-ray scattering and photoelectron spectroscopy in the hard X-ray range. J. Synchr. Rad..

[19] Céolin D (2013). Hard X-ray photoelectron spectroscopy on the GALAXIES beamline at the SOLEIL synchrotron. J. Electron. Spectros. Relat. Phenom..

[20] Simon M (2014). Atomic Auger Doppler effects upon emission of fast photoelectrons. Nature Comm..

[21] Céolin D (2015). Auger resonant-Raman study at the Ar K edge as probe of electronic-state-lifetime interferences. Phys. Rev. A.

[22] Kushawaha RK (2015). Auger resonant-Raman decay after Xe L-edge photoexcitation. Phys. Rev. A.

[23] Goldsztejn G (2016). Electronic state-lifetime interference in resonant Auger spectra: a tool to disentangle overlapping core-excited states. Phys. Chem. Chem. Phys..

[24] Püttner R (2015). Direct Observation of Double-Core-Hole Shake-Up States in Photoemission. Phys. Rev. Lett..

[25] Carniato S (2016). Photon-energy dependence of single-photon simultaneous core ionization and core excitation in CO_2_. Phys. Rev. A.

[26] Nakano M (2013). Near-Edge X-Ray Absorption Fine Structures Revealed in Core Ionization Photoelectron Spectroscopy. Phys. Rev. Lett..

[27] Reynaud C (1996). Double-core ionization and excitation above the sulphur K-edge in H2S, SO2 and SF6. J. Phys. B: At. Mol. Opt. Phys..

[28] Goldzstejn G (2016). Double-core-hole states in Ne: lifetime, post-collision interaction and spectral assignment. Phys. Rev. Lett..

[29] Siegbahn, K. *et al*. ESCA APPLIED TO FREEMOLECULES. N*orth-Holland Pub. Co*. (1969).

[30] Gelius U (1974). Recent progress in ESCA studies of gases. J. Electr. Spec. Rel. Phen..

[31] Ungier L, Thomas TD (1984). Resonance-Enhanced Shakeup in Near-Threshold Core Excitation of CO and N_2_. Phys. Rev. Lett..

[32] Carniato S (2015). Single photon simultaneous K-shell ionization and K-shell excitation. I. Theoretical model applied to the interpretation of experimental results on H_2_O. J. Chem. Phys..

[33] Wight GR, Brion CE (1974). K- and LII, III-Shell excitations in CS_2_ and COS by 2.5 keV electron impact. J. Electr. Spec. Rel. Phen..

[34] Adachi J, Kosugi N, Shigemasa E, Yagishita A (1996). Vibronic Couplings in the C 1s → *nsσ*_*g*_ Rydberg Excited States of CO_2_. J. Phys. Chem..

[35] Carniato S (2015). Single photon simultaneous K-shell ionization and K-shell excitation. II. Specificities of hollow nitrogen molecular ions. J. Chem. Phys..

[36] Perera RCC, LaVilla RE (1984). Molecular x-ray spectra: S-K*β* emission and K absorption spectra of SCO and CS_2_. J. Chem. Phys..

[37] Dehmer JL (1972). Evidence of effective potential barriers in the x-ray absorption spectra of molecules. J. Chem. Phys..

[38] Gianturco FA, Guidotti C, Lamanna U (1972). Electronic properties of sulfur hexafluoride. II. Molecular orbital interpretation of its x-ray absorption spectra. J. Chem. Phys..

[39] Tang R, Callaway J (1986). Electronic structure of sulfur hexafluoride. J. Chem. Phys..

[40] Nakamatsu H, Mukoyama T, Adachi H (1991). Theoretical x-ray absorption spectra of sulfur hexafluoride and hydrogen sulfide. J. Chem. Phys..

[41] Breinig M (1980). Atomic inner-shell level energies determined by absorption spectrometry with synchrotron radiation. Phys. Rev. A.

[42] King GC, Tronc M, Read FH, Bradford RC (1977). An investigation of the structure near the L_2,3_ edges of argon, the M_4,5_ edges of krypton and the N_4,5_ edges of xenon, using electron impact with high resolution. J. Phys. B: At. Mol. Opt. Phys..

[43] Ankerhold U, Esser B, von Busch F (1997). Decay of CS2 and OCS after sulphur 1s photoexcitation: II. Dissociation channels and kinematics. J. Phys. B: At. Mol. Opt. Phys..

[44] Andersson E (2010). Core-valence double photoionization of the CS_2_ molecule. J. Chem. Phys..

[45] Arneberg R, Ågren H, Malmquist P-Å, Svensson (1982). S. Multiple excitations in the core photoelectron spectrum of acetylene. Chem. Phys. Lett..

[46] Martin RL, Shirley DA (1976). Theory of core-level photoemission correlation state spectra. J. Chem. Phys..

[47] Tully JC, Berry RS, Dalton BJ (1968). Angular Distribution of Molecular Photoelectrons. Phys. Rev..

