# Supplementary material for: Cationic double K-hole pre-edge states of CS2 and SF6
Source: Sci Rep. 2017 Oct 17;7:13317. doi: 10.1038/s41598-017-13607-6 (PMC5645408; doi:10.1038/s41598-017-13607-6)
Supplement: Supplementary file 1 — Supplementary Materials [file 41598_2017_13607_MOESM1_ESM.pdf]

Supplementary Materials

Cationic double K-hole pre-edge states of CS_2 and SF_6

R. Feifel,[1] J.H.D. Eland,[1,2] S. Carniato,[3] P. Selles,[3] R. Püttner,[4] D. Koulentianos,[1,3] T. Marchenko,[3,5] L. Journel,[3,5] R. Guillemin,[3,5] G. Goldsztejn,[3,6] O. Travnikova,[3,5] I. Ismail,[3] B. Cunha de Miranda,[3] A.F. Lago,[7] D. Céolin,[5] P. Lablanquie,[3] F. Penent,[3] M.N. Piancastelli,[8,3] and M. Simon[3,5]

[1]*Department of Physics, University of Gothenburg, Origovägen 6B, SE-412 96 Gothenburg, Sweden*

[2]*Department of Chemistry, Physical and Theoretical Chemistry Laboratory, Oxford University, South Parks Road, Oxford OX1 3QZ, United Kingdom*

[3]*Sorbonne Universités, UPMC Univ Paris 06, CNRS, UMR 7614, Laboratoire de Chimie Physique-Matière et Rayonnement, 75005 Paris Cedex 05, France*

[4]*Fachbereich Physik, Freie Universität Berlin, Arnimallee 14, D-14195 Berlin, Germany*

[5]*Synchrotron SOLEIL, L'Orme des Merisiers, Saint-Aubin, BP 48, F-91192 Gif-sur-Yvette Cedex, France*

[6]*Max-Born-Institut, Max-Born-Strasse 2A, 12489 Berlin, Germany*

[7]*Centro de Ciências Naturais e Humanas, Universidade Federal do ABC (UFABC), Av. dos Estados, 5001, 09210-580, Santo André, SP, Brazil*

[8]*Department of Physics and Astronomy, Uppsala University, Box 516, SE-751 20 Uppsala, Sweden*

In what follows, further details on the calculations of the carbon K^{-2}V states in CS_2 are given in Supplementary Table S1 (= Table I below), and the vibrational analysis for SF_6 is described and summarized in Table S2 (= Table II below).

I. DETAILS ON THE CALCULATIONS OF THE K^{-2}V STATES

TABLE I. Characteristics of carbon K^{-2}V states of lowest binding energies in CS_2: Löwdin's populations corresponding to atomic orbitals of s, p, and d symmetry centered on the carbon atom; Term Values (TV) in eV (from DFT/B3LYP calculations). DFT/B3LYP DIP(K^{-2})=650.7 eV; DFT/B3LYP IP(K^{-1})=293.48 eV).

		K^{-2}V								K^{-1}V		
	MO	C*			S			DFT		Expt	DFT	
Region	assignment	s	p	d	s	p	d	Tv	$\sqrt{\langle r^2\rangle}$	Tv	Tv	$\sqrt{\langle r^2\rangle}$
(A)	π_u^*	0.00	0.19	0.00	0.00	0.38	0.02	16.09	1.89	7.00	7.50	1.85
(B)	$\mathbf{7}\sigma_g^*$	0.16	0.00	0.09	0.02	0.24	0.12	11.62	2.12	–	4.24	2.66
(C)	$\mathbf{6}\sigma_u^*$	0.00	0.21	0.00	0.01	0.18	0.20	10.11	2.21	2.50	2.90	4.00
(D)	$3s\sigma_g$	0.41	0.00	0.08	0.21	0.03	0.02	8.23	2.67	–	3.32	3.60

II. VIBRATIONAL ANALYSIS: CALCULATIONS OF FRANCK-CONDON FACTORS

A natural basis for the calculations of the Franck-Condon factors is the basis of normal coordinates. Here we assume that the ground state nuclear wavefunction can be written as a product of nuclear wavefunctions pertaining to normal vibrational modes (harmonic approximation).

Let us consider that the molecule has N internal degrees of freedom. For a normal mode with a bound potential energy well in the ground state and in the core ionized/excited final state (K^{-2}) the overlaps of the vibrational wave functions can easily be calculated in the case of harmonic potentials which is usually referred to as the linear coupling model. For the normal coordinate a, the displacement of the electronic initial state potential V_a^{gs} and the excited-state potential $V_a^{K^{-2}V}$ is

proportional to the gradient of the excited-state potential for the ground-state equilibrium geometry (see Ref. [1]).

For a steep slope (continuum of states) of the potential of the final electronic state, the overlap between the corresponding one-dimensional nuclear wave functions can be well approximated by a Gaussian function, so that the Franck-Condon factor (square of the overlap) for the K^{-2}V state, labeled "f", can be expressed as a function of the photoelectron energy ϵ_i^f associated with the continuum (Gaussian) distribution for mode i in the "f"-th K^{-2}V state as

$$FC(\epsilon_i^f) = \frac{2\sqrt{ln2}}{\Delta_i^f\sqrt{\pi}} exp\{-[4ln2(\frac{\epsilon_i^f}{\Delta_i^f})^2]\} \tag{1}$$

where Δ_i^f=2$\sqrt{ln2}$a$_i$F$_i^f$ is the width (FWHM) of the Franck-Condon distribution associated with the i-th normal mode. The partial derivative of the potential energy surface (PES) U along the i-th normal mode, taken at the equilibrium nuclear position Q_i^e of the ground state, is labeled by F_i^f and is hereafter referred to as a normal slope. The normal slope F_i^f is the partial derivative of the final-state potential energy surface calculated at the equilibrium nuclear positions Q_i^e of the ground state along the i-th normal mode:

$$F_i^f = \frac{\partial U}{\partial Q}|_e = \sqrt{\mu_i} \sum_{j=1}^{3N} \frac{A_j}{\sqrt{m_j}} \frac{\partial U}{\partial q_j}|_e \tag{2}$$

where A_j is the column vector corresponding to the symmetric stretching mode of the unitary transformation matrix between the mass weighted Cartesian and normal coordinates[2]. The width of the ground-state nuclear wavefunction along the i-th normal mode, hereafter referred to as the normal width, equals $a_i = \sqrt{\hbar/(\mu_i\omega_{ki})}$, μ_i and ω_{ki} being the reduced mass and the pulsation of the i-th normal mode, respectively. The final-state potential energy curve was approximated in the region close to the equilibrium geometry by a linear function. The gradients along the normal modes were calculated within the GAMESS(US) package at the DFT level of theory, using the Becke three-parameter hybrid exchange and the Lee-Yang-Parr (B3LYP) gradient-corrected correlation functional. In the case of few large steep slopes along N_m different (normal coordinate) Q_k directions, the full width can be estimated as follow:

$$\Delta_{tot}^{FC} = \sqrt{\sum_{j=1}^{N_m} (\Delta_j^f)^2} \tag{3}$$

TABLE II. Comparison between theoretical DFT/B3LYP and experimental vibrational frequencies (in cm^{-1}) of SF_6. The most important (calculated) individual Gradient F_i (in eV/Å) for S-$K^{-2}(6a_g^*)$ and S-$K^{-2}(6t_{1u}^*)$ and F-$K^{-2}(14a_1^*)$ F-$K^{-2}(15a_1^*)$ F-$K^{-2}(10e^*)$ final states are given. In parenthesis, Δ is the full width at half maximum of the Franck-Condon profile for the i-th mode. Δ_{tot}^{FC} is the full width at half maximum of the Franck-Condon profile.

Vibrational mode	Vibrational frequencies		Gradient (F_i)				
	Ground state		S^*F_6		SF^*_6		
	ω_i^{DFT}	ω_i^{Expt}	$K^{-2}(V=6a_{1g}^*)$	$K^{-2}(V=6t_{1u}^*)$	$K^{-2}(V=14a_1^*)$	$K^{-2}(V=15a_1^*)$	$K^{-2}(V=10e^*)$
Antisym. stretch (E_g)	604	643	–	10.93 (0.98)	15.10 (1.36)	18.50 (1.67)	10.97 (0.99)
Sym. stretch (A_{1g})	731	774	-10.57(0.87)	-11.80 (0.97)	-15.66 (1.28)	-18.86 (1.55)	-14.81 (1.21)
Sym. stretch (F_{1u})	891	948	–	–	40.4 (2.58)	30.25 (1.93)	31.00 (1.98)
Δ_{tot}^{FC}			0.87	1.38	3.20	3.00	2.52

[1] Ilakovac, V., Carniato, S., Gallet, J.-J., Kukk, E., Horvatic, D. & Ilakovac, A. Vibrations of acrylonitrile in N 1s excited states. *Phys. Rev. A* **77**, 012516-1 - 012516-9 (2008).

[2] Carniato, S. *et al.* Single photon simultaneous K-shell ionization and K-shell excitation. I. Theoretical model applied to the interpretation of experimental results on H_2O *J. Chem. Phys.* **142**, 014307-1 - 014307-10 (2015).
